# Comprehensive geriatric assessment for older orthopedic patients and analysis of risk factors for postoperative complications

**DOI:** 10.1186/s12877-022-03328-5

**Published:** 2022-08-04

**Authors:** Chao Kong, Yanhong Zhang, Chaodong Wang, Peng Wang, Xiangyu Li, Wei Wang, Yu Wang, Jianghua Shen, Xiaoyi Ren, Tianlong Wang, Guoguang Zhao, Shibao Lu

**Affiliations:** 1 Department of Orthopedics, Xuanwu Hospital of Capital Medical University, 45 Changchun Street, Xicheng, Beijing, 100053 People’s Republic of China; 2 National Geriatric Disease Research Center, Xuanwu Hospital of Capital Medical University, 45 Changchun Street, Xicheng, Beijing, 100053 People’s Republic of China; 3 Beijing Municipal Geriatric Medical Research Center, Xuanwu Hospital of Capital Medical University, 45 Changchun Street, Xicheng, Beijing, 100053 People’s Republic of China

**Keywords:** Comprehensive geriatric assessment, Older people; risk factor; Postoperative complication, Orthopedic surgery

## Abstract

**Background:**

The comprehensive geriatric assessment (CGA) has been proposed as a supplementary tool to reduce perioperative complications of geriatric patients, however there is no universally accepted standardization of CGA for orthopedic surgery. In this study, a novel CGA strategy was applied to evaluate the conditions of older patients undergoing orthopedic surgery from a broad view and to identify potential risk factors for postoperative complications.

**Methods:**

A prospective cohort study was conducted from March 2019 to December 2020.The study enrolled patients (age > 75 years) for elective or confined orthopedic surgery. All patients were treated by a multidisciplinary team. A structured CGA was conducted to identify high-risk older patients and to facilitate coordinated multidisciplinary team care by a geriatric team. The basic patient characteristics, CGA results, postoperative complication and mortality rates were collected. Multivariate logistic regression analysis was used to identify risk factors for postoperative complications.

**Results:**

A total of 214 patients with an age of 81.07 ± 4.78 (range, 75–100) years were prospectively enrolled in this study. In total, 66 (30.8%) complications were registered, including one death from myocardial infarction (mortality rate, 0.5%). Poor Activities of Daily Living (ADL) and Instrumental Activities of Daily Living (IADL) were accompanied by frailty, worse perioperative risk, pain, and nutritional status. Poor ADL was also associated with higher risks of falling, polypharmacy, and cardiac and respiration complications. Poor IADL was associated with a higher risk of cardiac and respiration complications. Higher stroke risk was accompanied by higher risks of cardiac complications, delirium, and hemorrhage. Worse American Society of Anesthesiologists (ASA) score was associated with worse ADL, IADL, frailty, and higher delirium risk. Multivariate logistic regression analysis showed that spinal fusion (odds ratio [OR], 0.73; 95% confidence interval [CI], 0.65 to 0.83; *p* = 0.0214), blood loss(OR, 1.68; 95% CI, 1.31 to 2.01; *p* = 0.0168), ADL (severe dysfunction or worse) (OR, 1.45; 95% CI, 1.16 to 1.81; *p* = 0.0413), IADL (serious dependence) (OR, 1.08; 95% CI, 1.33 to 1.63; *p* = 0.0436), renal function (chronic kidney disease (CKD) ≥ stage 3a) (OR, 2.01; 95% CI, 1.54 to 2.55; *p* = 0.0133), and malnutrition(OR, 2.11; 95% CI, 1.74 to 2.56; *p* = 0.0101) were independent risk factors for postoperative complications.

**Conclusion:**

The CGA process reduces patient mortality and increases safety in older orthopedic surgery patients. Spinal fusion, blood loss, ADL (severe dysfunction or worse), IADL (serious dependence), renal function (CKD ≥ stage 3a) and nutrition mini nutritional assessment (MNA) (malnourished) were independent risk factors of postoperative complications following orthopaedic surgery in older patients.

**Supplementary Information:**

The online version contains supplementary material available at 10.1186/s12877-022-03328-5.

## Background

Based on statistics from the United States Census Bureau [1], the world’s population aged 65 years and over is predicted to reach 1.6 billion by the year 2050. In China, the population aged 65 years and over is projected to reach 500 million by the year 2050 according to the sixth population census published by the National Bureau of Statistics [2]. With the worldwide increase in the aged population, the demand for surgical procedures and total expenditure for Medicare is also expected to rise [3]. China's aging trend suggests that the population aging level is very likely to continue to intensify and affects the medical insurance fund balance in the future [4]. As part of that overall surge in cost, orthopedic surgeons will face particular challenges in caring for this aging patient population.

The World Health Organization defines late older people individuals as those 75 years or older. It’s recognized that geriatric patients are accompanied by multiple chronic illnesses that limit their functional capacity and recovery [5]. Moreover, non-disease-associated problems, such as frailty, polypharmacy, decreased functional dependence, poor nutritional status, and cognitive decline may also complicate surgical procedures and postoperative recovery [6, 7]. Despite the advances in surgical and anesthetic techniques, geriatric patients have higher perioperative complications and mortality compared to adults [7, 8]. In order to provide optimal care for the older surgical patients, it is essential to conduct a thorough assessment of the individual’s health status and a plan of care during the perioperative period to identify and address deficits.

Comprehensive geriatric assessment (CGA) is a multidimensional interdisciplinary diagnostic process to determine the medical, psychosocial, and functional capabilities of a frail older patient and develop a coordinated and integrated plan for maximizing overall health with aging [9, 10]. As the gold standard for in-hospital care of geriatric patients, the effectiveness of CGA has been confirmed by many studies [11, 12]. Despite the benefits of the current CGA, there are still some limitations. First, there is no universally accepted standardization in the assessment. The focused domains and assessment tools differ among studies [13–15]. Second, there is still uncertainty about identifying and targeting suitable patients who are most likely to benefit from CGA [11].

Based on these considerations, the China’s National Center for Clinical Medicine of Geriatric Diseases assembled a 23-member multidisciplinary panel from 10 domains. After one year, a novel CGA tool was established based on a focused and structured literature review. This new CGA tool consists of two parts: primary screening and multidisciplinary assessment (within 21 domains). The aim of this study was to describe our new CGA tool and its impact on health outcomes in the postoperative period among geriatric patients undergoing orthopedic surgery.

## Methods

### Overview

The protocol of this prospective study conducted from March 2019 to December 2020 was approved by the regional medical ethics committee and registered in Chinese clinical trial registry (ChiCTR1800020363). The inclusion criteria were: 1) aged ≥ 75 years with an expected preoperative time of ≥ 2 days, and 2) patients scheduled for elective or confined orthopedic surgery. Exclusion criteria were patients who refused participation and study-enrolled patients who were recommended to conservative treatment or minimally invasive surgery because they could not be optimized in a short-term preoperative optimization after preoperative assessments. All patients provided informed consent before surgery.

### CGA

**Fig. 1:**
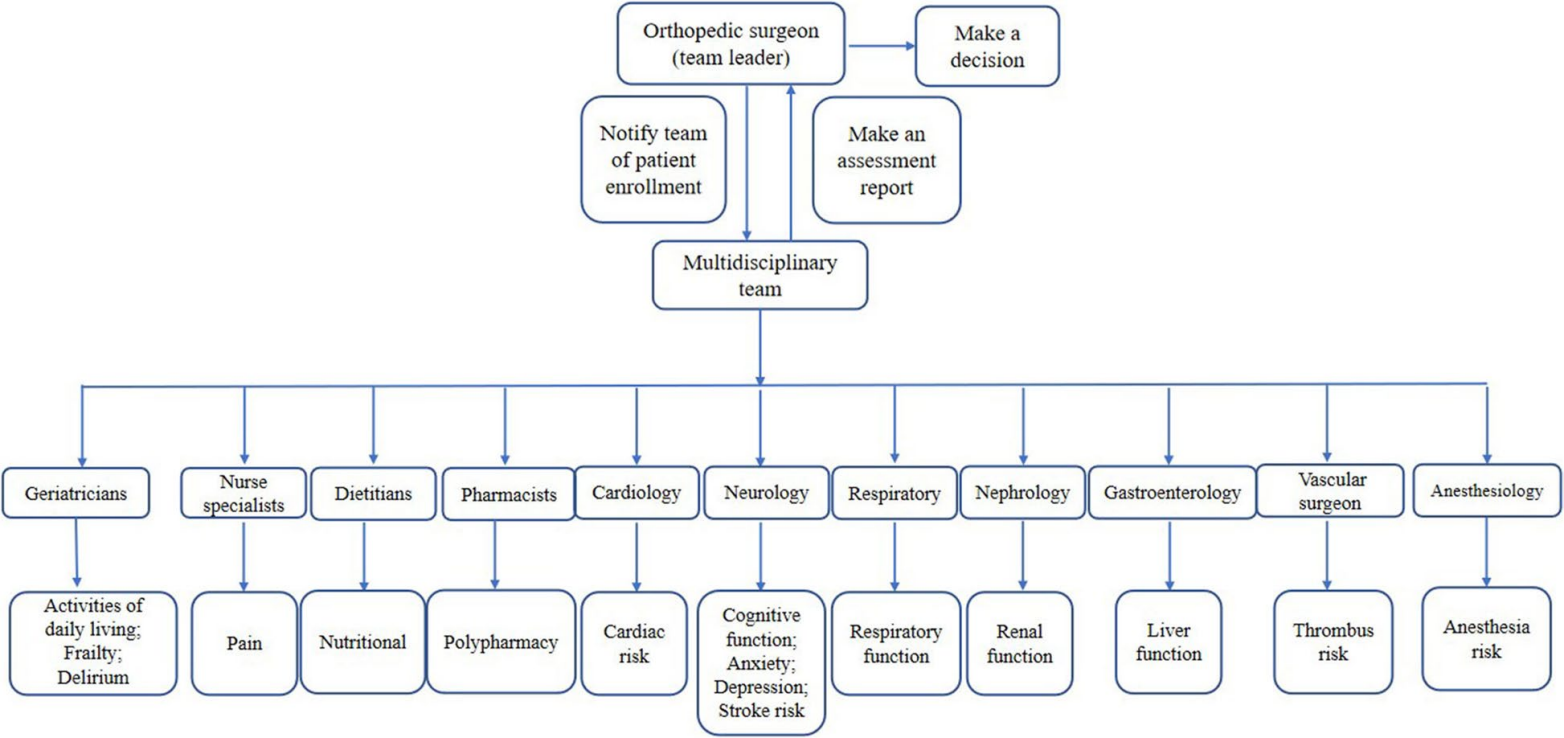
Different CGA team members tasks specific to this study

All patients were enrolled and treated with the help of a perioperative multidisciplinary team. Preparing patients for orthopedics surgery were seen in the orthopedics department for one-station interprofessional perioperative evaluation and care coordination. The multidisciplinary team consists of geriatricians, nurse specialists, vascular surgeon, dietitians, pharmacists, and physicians from the departments of cardiology, anesthesiology, neurology, respiratory, nephrology and gastroenterology. All members of the multidisciplinary team had more than 5 years of clinical experience and were specially trained for scale assessment. The multidisciplinary team actively engaged family members of patients with cognitive impairment to join the preoperative risk assessment. Preoperative multidisciplinary evaluation will be completed within 48 h after the patient is admitted to hospital, multidisciplinary assessment included 21 specific forms covering activities of daily living, nutritional status, cardiac function, pulmonary function, renal function, hepatic function, frailty, cognition, depression, delirium and medications, our multidisciplinary assessment model is shown in Fig. 1. The multidisciplinary team offered recommendations for risk-reducing strategies in the preoperative and postoperative periods by Aged Patient Perioperative Longitudinal Evaluation-Multidisciplinary Decision-Making System (APPLE-MDT) [16]. To facilitate the implementation of recommendations made before surgery, the multidisciplinary team collaborated with the surgical teams to conduct preoperative optimization. During the postoperative period, the multidisciplinary team followed patients conduct evaluation complications and provide rehabilitation guidance before discharge. The multidisciplinary team was led by the orthopedic surgeons, in the event of disagreement among the multidisciplinary team, the orthopedic surgeon leader was responsible for making decisions.

### Data collected and outcome measures

After evaluation, data were collected by the multidisciplinary team from patient-completed questionnaires uploaded to the APPLE-MDT by themselves, and basic information of patient were collected by nurses abstracted from the clinic note into APPLE-MDT.

For preoperative assessment, each specialist will go to the patient's ward for interview evaluation, and upload the evaluation results to the APPLE system by themselves, and basic information about the patient was collected by nurses abstracted from the clinic note into APPLE-MDT. After the system automatically summarizes, the orthopedic surgeons informed the patient of the multidisciplinary assessment conclusion.

All clinical data were prospectively collected within 48 h after the patient is admitted to hospital by a doctor and a nurse independent of this study. In this study, data consisted of two parts: primary screening and multidisciplinary assessment.

The primary screening included patient characteristics, routine blood tests, nutrition, electrolyte, blood glucose, coagulation index, stress echocardiography, electrocardiogram, pulmonary function tests, transcranial color doppler, and deep vein ultrasound of the lower extremity. The multidisciplinary assessment was conducted with the use of specific assessment forms. The evaluation relevant scale in this study adopts the recommendation of Chinese expert consensus on the perioperative multidisciplinary evaluation of older patients with spinal surgery [17]. Functional status was determined based on Barthel index Activities of Daily Living (ADL) and functional activities questionary Instrumental Activities of Daily Living (IADL) [18], functional were directly assessed by geriatrician. ADL included the fundamental skills typically needed to manage basic physical needs, comprising the following areas: grooming/personal hygiene, dressing, toileting/continence, transferring/ambulating, and eating. Mild dysfunction with a score between 75 and 95 point; Moderate dysfunction between 50 and 70 points; Severe dysfunction between 25 and 45 points; Less than 20 points for extremely severe dysfunction. IADL included more complex activities related to independent living in the community. Mild dependence with a score between 6and 7 point; Moderate dependence between 3 and 5 points; Less than 2 points for serious dependence. Frailty evaluation using the Fried Assessment. Nutritional status was evaluated using the mini nutritional assessment (MNA) [19]. Pain using the visual analogue scale (VAS). Renal function using the chronic kidney disease (CKD) stage classification, the staging of CKD is mainly based on the severity of the glomerular filtration rate (GFR) [20], and is divided into five stages. The common stages are as follows: stage 1: the GFR level is above 90 ml/(min); stage 2: the GFR level is 60-89 ml/(min); stage 3a: the GFR level is 45-59 ml/(min); stage 3b: the GFR level is 30-44 ml/(min); stage 4: the GFR level is 15-29 ml/(min); stage 5: the GFR level is less than15ml/(min). Liver function using the modified Child–Pugh classification [21], the Child–Pugh scoring system was designed to predict mortality in patients, to guide the selection of patients who would benefit from elective surgery, it broke down patients into three categories: A—good hepatic function, B—moderately impaired hepatic function, and C—advanced hepatic dysfunction. Respiratory function using the Arozullah postoperative respiratory failure risk score [22]. Thrombus risk evaluation using the Caprini score [23]. Cardiac risk evaluation using Lee’s revised cardiac risk index [24]. Anesthesia risk using the American Society of Anesthesiologists (ASA) scale [25]. Cognitive function using the Montreal Cognitive Assessment (MoCA) and mini mental status examination (MMSE) [26], cognitive impairment assessment by geriatrician. MoCA was developed as a brief screening instrument for mild cognitive impairment. The MoCA is divided into 7 subscores: visuospatial/executive (5 points); naming (3 points); memory (5 points for delayed recall); attention (6 points); language (3 points); abstraction (2 points); and orientation (6 points). One point is added if the subject has ≤ 12 years of education, and a score ≤ 25 was found to be the optimal cutoff point for a diagnosis of cognitive. Mild cognitive impairment with a score between 18 and 25 points; Moderate cognitive impairment between 10 and 17 points; Less than 10 points for severe cognitive impairment. Anxiety and depression assessment by geriatrician. Anxiety using the Zung’s Self-Rating anxiety scale (SAS) [27], the SAS consists of 20 self-report items on anxiety symptom, anxiety symptom was defined if the SAS score was ≥ 50 points. Depression evaluation using Zung’s Self-Rating Depression Scale (SDS) [28], the SDS consists of 20 self-report items on depression symptom, depression symptom was defined if the SDS score was ≥ 53 points. And stroke risk using the Revised Framingham Stroke Risk Profile and the Essen Stroke Risk Score [29, 30]. Perioperative risks of massive hemorrhage, falling, delirium, and polypharmacy were also evaluated. Postoperative complications or mortality were defined as complications or death within 30 days after surgery.

### Statistical analysis

Continuous variables are presented as the mean ± standard deviation (SD) and analyzed with the independent-sample *t*-test. Categorical variables are presented as percentages and analyzed with the chi-square test. Spearman correlation analysis was used to analyze the association among different CGA domains. Multivariate logistic regression analysis was used to identify risk factors for postoperative complications. A probability (*p*) value of < 0.05 was considered statistically significant. All statistical analyses were performed using SPSS software (version 18.0; SPSS Inc., Chicago, IL, USA).

## Results

**Fig. 2:**
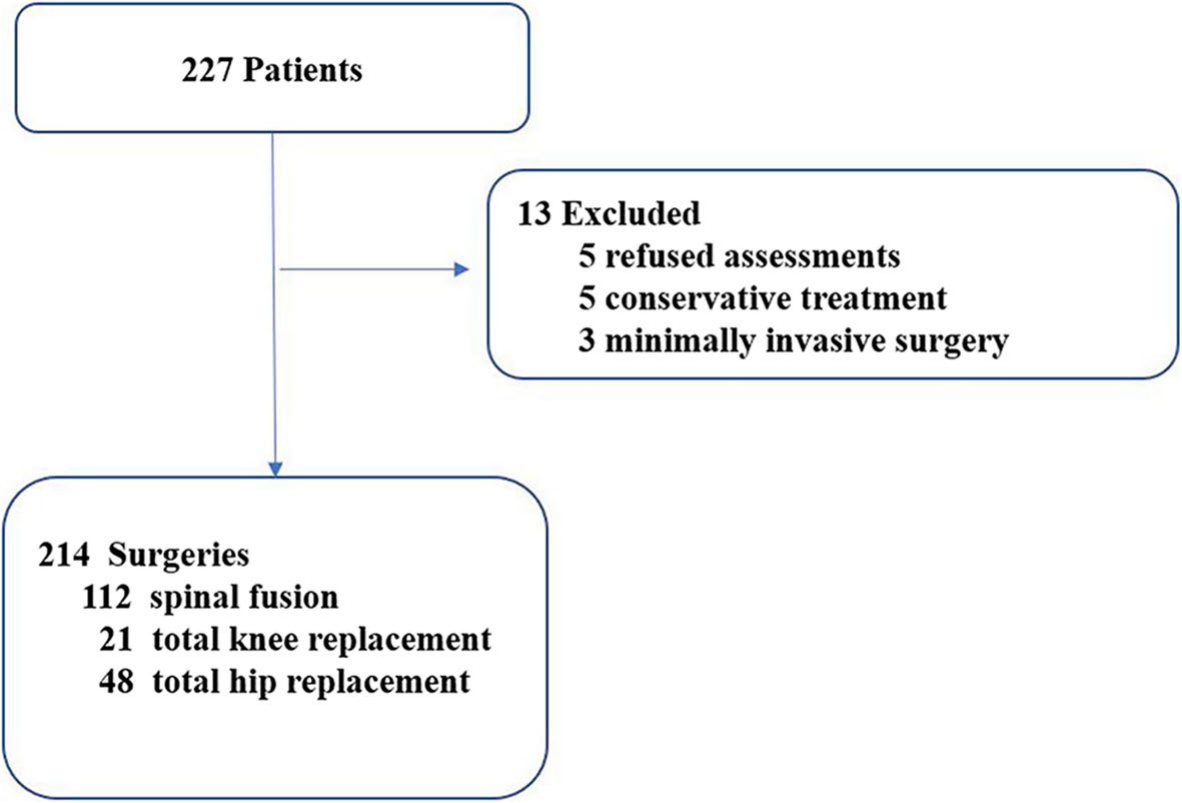
Patient recruitment in our program

Figure 2 shows the total number of patients enrolled in our program. There were five patients who refused participation. After preoperative assessments, five patients were recommended for conservative treatment and three patients underwent minimally invasive surgery due to the high risk of spine open surgery or who cannot be improved by short-term preoperative optimization were excluded in this study. A total of 214 patients with an average age of 81.07 ± 4.78 (range, 75–100) years were included for analysis. The demographic and clinical characteristics of all patients are listed in Table 1. Of all patients, 112 (52.3%) underwent spinal fusion, 54 (25.2%) had total knee replacement, and 48 (22.4%) had total hip replacement, for a complete consort diagram of included patients, please see Fig. 2. The most common comorbidity was hypertension, which was present preoperatively in 65.4% of the patients. Other common comorbidities were diabetes (24.8%), coronary heart disease (21.5%), cerebral infarction (14.3%), arrhythmia (6.3%), renal insufficiency (3.1%), and chronic obstructive pulmonary disease (3.5%). In addition, 26.6% of the patients had one comorbidity, 23.8% had two, 21% had three, 11.7% had more than three, and 16.8% had none. The mean number of comorbidities was 2.5 per patient (SD, 1.7).

**Table 1:** Patient demographic and clinical characteristics

	Patients (*N* = 214)	
Characteristic	No. (%)	
Age, mean ± SD, y	81.07 ± 4.78	
BMI, mean ± SD	25.09 ± 4.17	
Male sex	72(33.6)	
Smoking (yes/no)	22(10.3)	
Alcohol (yes/no)	16(7.5)	
Comorbidities		
0	36(16.8)	
1	56(26.2)	
2	51(23.8)	
3	45(21)	
> 3	26(12.1)	
ASA scale		
2	39(18.2)	
3	163(76.2)	
4	12(5.6)	
Type of surgery		
-Spinal fusion	112(52.3)	
-Total knee replacement	54(25.2)	
-Total hip replacement	48(22.4)	
Surgical duration, mean ± SD, min	151.50 ± 86.74	
Blood loss, mean ± SD, ml	285.53 ± 316.34	

Abbreviations: *ASA* American Society of Anesthesiologists, *BMI* Body mass index, *SD* Standard deviation

### Postoperative outcomes and complications

One patient died of myocardial infarction 7 days after surgery, resulting in a mortality rate of 0.5%. A total number of 66 (30.8%) complications were registered. Implant-related complications are not discussed in this article. The most frequent postoperative complications were hypoproteinemia (albumin ≤ 30 g/L) (9.8%), followed by wound infection (3.7%), pneumonia (2.8%), urinary tract infection (2.3%), delirium (1.9%), transient arrhythmia (1.4%), pressure sores (1.4%), cerebral infarction (1.4%), myocardial infarction (1.4%), ileus (0.9%), gastrointestinal tract bleeding (0.9%), respiratory failure (0.9%), deep vein thrombosis (0.5%), cardiac failure (0.5%), and pulmonary embolus (0.5%). The mean hospital stay was 13.45 ± 7.46 days.

Comparisons of demographic and clinical characteristics between patients with and without complications are shown in Table 2. Patients undergoing spinal fusion and those with more blood loss tended to have higher risks of postoperative complications (*p* ≤ 0.002).

**Table 2:** Comparisons of demographic and clinical characteristics between patients with and without complications

Characteristic	With complications (*n* = 66)	Without complications (*n* = 148)	*p*
Mean age	80.94 ± 4.57	81.14 ± 5.14	0.741
Male sex (n/%)	18 (27.3%)	54 (36.5%)	0.188
BMI	24.83 ± 4.48	25.21 ± 3.97	0.684
Smoking (yes/%)	10 (15.2%)	12 (8.1%)	0.117
Alcohol (yes/%)	7 (10.6%)	9 (6.1%)	0.245
Comorbidities			0.360
no	10	26	
1	13	43	
2	15	36	
3	17	28	
> 3	11	15	
ASA scale			0.289
2	13	26	
3	47	116	
4	6	6	
Type of surgery			< 0.001 ^*^
-Spinal fusion	44	68	
-Total knee replacement	5	49	
-Total hip replacement	17	31	
Surgical duration (min)	160.46 ± 91.62	147.72 ± 81.60	0.475
Blood loss (ml)	438.65 ± 479.14	217.24 ± 252.75	0.002 ^*^

Abbreviations: *ASA* American Society of Anesthesiologists, *BMI* Body mass index

^*^Significant differences between groups

### Preoperative CGA results

All patients received a thorough CGA with multidisciplinary assessment completed within 48 h. About 63.1% of all patients had moderate or severe pain before surgery, 28.5% had poor ADL/IADL (severe or extremely severe dysfunction and were seriously dependent), 66.4% were frail, and 14% were malnourished before surgery. Most patients had satisfactory perioperative blood glucose control (89.25%) and normal thyroid function (86.92%). Renal function was stage 3a or worse in 101 (47.2%) patients, while 93.93% had normal liver function, as determined with the modified Child–Pugh classification.

Overall, 75 patients (35.05%) were at high risk for cardiac complications, 196 (91.59%) were at extremely high risk for thrombus, 176 patients (82.24%) had a high risk of massive hemorrhage. Respiration risk distributions were 0.5% (49 patients, 22.89%), 1.8% (88 patients, 41.12%), 4.2% (68 patients, 31.78%), and 10.1% (9 patients, 4.21%). In addition, the risk of stroke was high in 42 patients (19.63%), delirium in 16 (7.48%), falling in 11 (5.14%), and polypharmacy in 160 (74.77%).

Mild cognitive impairment was identified in 113 patients (52.8%). Five patients (2.34%) had anxiety and 5 (2.34%) had depression before surgery. The distribution of perioperative ASA scale was: grade II (42 patients, 19.63%), grade III (156 patients, 72.9%), and grade IV (16 patients, 7.47%).

Spearman correlation analysis was used to analyze the association among different evaluation items. Poor ADL and IADL were accompanied by frailty and worse ASA, VAS, and nutritional status. Poor ADL was also associated with higher risks of falling, polypharmacy, cardiac complications, and respiration complications. Poor IADL was associated with higher risks of cardiac and respiration complications. Higher stroke risk was accompanied with higher risks of cardiac complications, delirium, and hemorrhage. A poor ASA score was associated with worse ADL, IADL, frailty, and higher delirium risk. Other associations of CGA results are shown in a correlation matrix presented in Table 3.

**Table 3:** Spearman correlation matrix of CGA results

	IADL	VAS	Frailty	Nutrition	Renal function	Liver function	Cognition	Anxiety	Depression	Stroke risk	Falling risk	Polypharmacy risk	Cardiac risk	Respiration risk	Delirium risk	Caprini thrombus risk	Hemorrhage risk	ASA
ADL	< 0.001*	< 0.001*	< 0.001*	< 0.001*	0.206	0.461	0.414	0.245	0.228	0.064	0.014*	0.026*	0.017*	< 0.001*	0.325	0.124	0.388	0.008*
IADL		< 0.001*	< 0.001*	< 0.001*	0.358	0.691	0.551	0.167	0.211	0.138	0.055	0.125	0.031*	0.004*	0.368	0.166	0.443	0.014*
VAS			< 0.001*	0.173	0.431	0.824	0.779	0.041*	0.036*	0.959	0.214	0.112	0.002*	0.034*	0.942	0.075	0.821	0.385
Frailty				< 0.001*	0.185	0.891	0.674	0.314	0.225	0.255	0.143	0.434	0.617	0.015*	0.299	0.186	0.808	0.033*
Nutrition					0.026*	0.779	0.814	0.457	0.613	0.413	0.533	0.381	0.851	0.089	0.144	0.863	0.723	0.053
Renal function						0.481	0.578	0.781	0.814	0.871	0.311	0.468	0.103	0.13	0.208	0.253	< 0.001*	0.264
Liver function							0.331	0.654	0.588	0.482	0.889	0.033*	0.132	0.228	0.069	0.097	0.118	0.475
Cognition								0.441	0.523	0.142	0.663	0.459	0.253	0.583	0.177	0.201	0.329	0.344
Anxiety									0.012*	0.412	0.491	0.101	0.389	0.407	0.227	0.761	0.631	0.712
Depression										0.389	0.518	0.189	0.411	0.553	0.308	0.80	0.503	0.538
Stroke risk											0.668	0.378	0.023*	0.175	0.006*	0.956	0.015*	0.217
Fall risk												0.703	0.318	0.377	0.199	0.202	0.566	0.289
Polypharmacy risk													0.289	0.47	0.203	0.338	0.414	0.51
Cardiac risk														0.006*	0.736	0.173	0.693	0.063
Respiration risk															0.077	0.135	0.262	0.084
Delirium risk																0.173	0.693	< 0.001*

Abbreviations: *ADL* Activities of Daily Living, *ASA* American Society of Anesthesiologists, *IADL* Instrumental Activities of Daily Living, *VAS* Visual analogue scale

^*^Significant differences were found

### Univariate analysis of potential risk factors related to complications

The associations between postoperative complications and domains of the CGA are listed in Table 4 (other domains are listed in supplementary file). Univariate analysis showed that six domains were significantly associated with postoperative complications. Patients with dependent ADL, dependent IADL, worse renal function, worse nutritional status, cognitive impairment, and low ASA scores tended to have higher risk of postoperative complications (*p* ≤ 0.0442).

**Table 4:** Univariate Analysis of Preoperative CGA Related to Postoperative Complications

	Number (with any complication, %)	*p*^a^
**ADL**		**0.0296 **^**b**^
Independent	21(19.0%)	
Mild dysfunction	58(23.7%)	
Moderate dysfunction	75(28.4%)	
Severe dysfunction	45(39.5%)	
Extremely severe dysfunction	15(58.8%)	
**IADL**		**0.0354 **^**b**^
Normal	34(17.6%)	
Mild dependence	39(25.6%)	
Moderate dependence	80(28.8%)	
Serious dependence	61(44.3%)	
**Nutrition (MNA)**		**0.0442 **^**b**^
Normal	114(23.7%)	
At risk of malnutrition	70(37.1%)	
Malnourished	30(43.3%)	
**Cognitive impairment**		**0.0052 **^**b**^
No impairment	101(19.8%)	
Mild impairment	113(40.7%)	
**Renal function**		**0.0134 **^**b**^
Normal	7(0%)	
Stage 1	22(22.7%)	
Stage 2	84(23.8%)	
Stage 3a	69(44.9%)	
Stage 3b	26(38.5%)	
Stage 4	4(0%)	
Stage 5	2(0%)	
**ASA score**		**0.0376 **^**b**^
Grade II	39(16.7%)	
Grade III	163(35.3%)	
Grade IV	12(25.0%)	

Abbreviations: *ADL* Activities of Daily Living, *ASA* American Society of Anesthesiologists, *IADL* Instrumental Activities of Daily Living, *MNA* Mini nutritional assessment, *VAS* Visual analogue scale;

^a^Kruskal–Wallis test

^b^Significant differences among groups

### Multivariate analysis of significant univariate factors

**Fig. 3:**
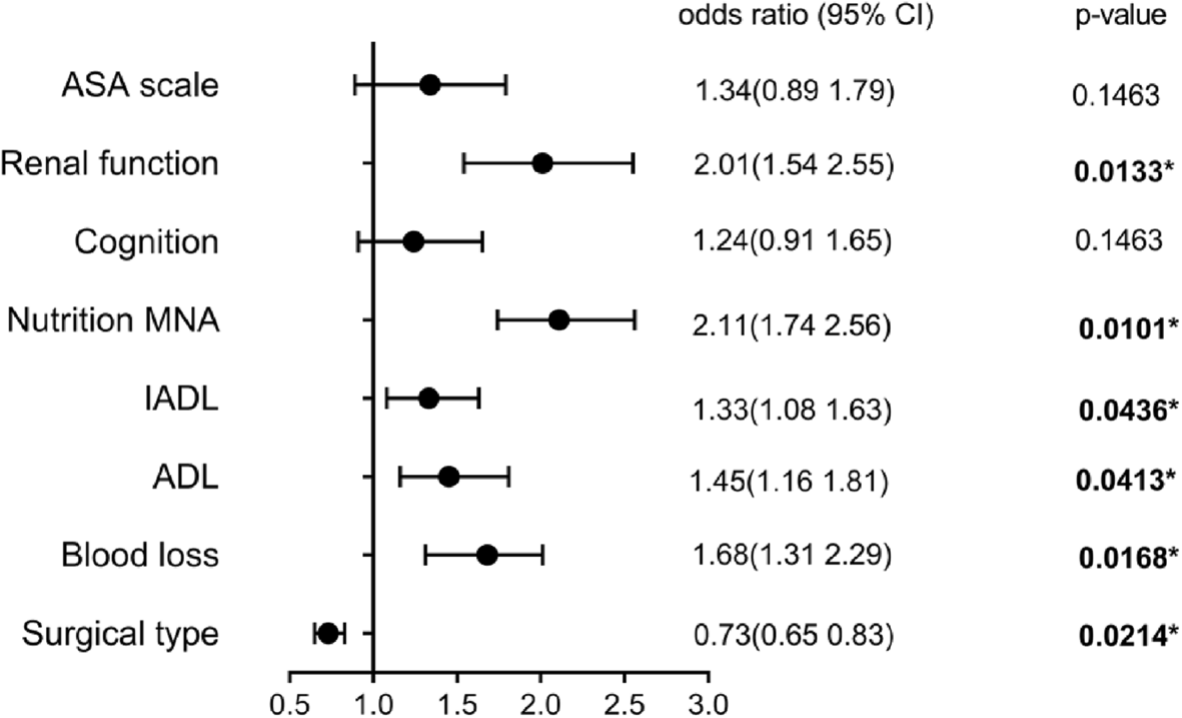
Forest plot of the relationships between preoperative parameters and postoperative complications

Eight potential risk factors (i.e., type of surgery, blood loss, ADL, IADL, nutritional status, renal function, ASA score, and cognitive impairment) were included for logistic regression analysis. Multivariate analysis showed that spinal fusion (odds ratio [OR], 0.73; 95% confidence interval [CI], 0.65 to 0.83; *p* = 0.0214), blood loss (OR, 1.68; 95% CI, 1.31 to 2.01; *p* = 0.0168), ADL (severe dysfunction or worse) (OR, 1.45; 95% CI, 1.16 to 1.81; *p* = 0.0413), IADL (serious dependence) (OR, 1.08; 95% CI, 1.33 to 1.63; *p* = 0.0436), renal function (CKD ≥ stage 3a) (OR, 2.01; 95% CI, 1.54 to 2.55; *p* = 0.0133), and malnutrition (MNA) (OR, 2.11; 95% CI, 1.74 to 2.56; *p* = 0.0101) were independent risk factors for postoperative complications (Fig. 3).

## Discussion

As the population continues to age, the demand for surgical services perioperative care will significantly increase. Surgical decisions for older and frailer patients require not only fitness for the procedure itself, but also the consideration of other factors, such as comorbidities, disability, cognitive impairment, and nutritional status. Many studies have described proactive and coordinated multidisciplinary assessment for older patients undergoing surgery [31, 32]. However, these studies employed limited evaluation domains and few have focused on elective orthopedic surgery, especially spine surgery.

In this study, a novel CGA preoperative assessment was adopted that incorporated 21 specific forms from 10 domains. A thorough manifestation of geriatric conditions was shown in this study. Most patients in this cohort study underwent elective surgery with the aim of improving the quality of life, which explains the high prevalence of severe pain and dependence in daily life. One characteristic of the aging body is a reduction of physiological reserve, or frailty. Frailty, is a geriatric syndrome resulting from the declines of multiple physiological systems, characterized by malnutrition, exercise intolerance, and lower gaits peed has become one of the biggest challenges in facilitating healthy aging [33]. More than 66% of all patients in this study were frail, which was higher than those reported in Fried’s study [34]. Their patients were younger with less pain and dysfunction, which may have influenced the results. The prevalence of malnutrition among the older people was 22.8%, with considerable differences between the settings studied (rehabilitation, 50.5%; hospital, 38.7%; nursing home, 13.8%; community, 5.8%) [35]. The proportion of malnutrition in this study was merely 14%, much lower than in the general older population. As a possible explanation, unlike the Western culture of older living independently, most Chinese older people live with their children and may more likely to eat healthy and regularly. In addition, there is currently a lack of authoritative reports on the prevalence of malnutrition among the older people in China. The prevalence of cognitive impairment among those aged ≥ 60 years in the Swedish in 2019 was reportedly 22.6% [36]. In China, an estimated 20% of the population older than 65 years reportedly have cognitive impairment [37]. The prevalence of cognitive impairment reported in this study was higher than in a population of healthy older, but lower than older patients undergoing surgery for spinal deformities [38].

Though numerous studies have described preoperative CGA results, few have examined the inner relationships among different evaluation items. In this study, we established a correlation matrix of CGA results using Spearman correlation analysis. As shown in Table 4, poor ADL and IADL were accompanied by frailty and worse ASA, VAS, and nutritional status. Higher stroke risk was accompanied with higher risks for cardiac complications, delirium, and hemorrhage. Worse ASA scores were associated with worse ADL and IADL, as well as frailty and higher delirium risk. Moreover, the cumulative effect of evaluation results should be interpreted with caution. Makary et al. [39] found that frailty was a better predictor of outcomes when combined with other evaluation forms. Therefore, before surgery or intervention management, it is necessary to make sure that all preoperative evaluation results are handled as a whole,.

The overall mortality and complication rates in this study were 0.5% and 30.8%, respectively. The mortality rate was relatively lower, while the complication rate was consistent with other similar studies. Compared with other geriatric orthopedic studies, the mortality rate of patients in our study was lower [40–42]. The main reason is that the patients included in this study were all patients undergoing elective surgery, and there were no patients with hip fractures, which are a major cause of mortality and disability in frail older adults. And all patients received our complete evaluation and optimization before surgery is also an important factor in reducing the mortality. In the Surgical Quality Improvement Program database of the American College of Surgeons National, including standardized preoperative, intraoperative, and 30-day postoperative data points, the morbidity and mortality rates of a total of 7,696 surgical procedures were 28% and 2.3%, respectively [43].

The purpose of a CGA preoperative assessment is to identify patients with an elevated risk for poor surgical outcomes. Age, by itself, does not increase surgical risk; rather, the increased prevalence of chronic disease and the deterioration of the organ function associated with aging might increase the risk of postoperative complications. By taking the CGA assessment, the complications of orthopedic surgery in the older patients are not significantly increased, and the low surgical complications can effectively accelerate postoperative recovery, reduce hospitalization days, and reduce the medical cost burden on families and society. In this study, four CGA domains (i.e., ADL, IADL, renal function, and nutritional status) were significantly associated with increased risks of postoperative complications.

Functional status is known to be a significant predictor of postoperative outcomes, poor functional status was a risk factor for postoperative complications [44, 45]. In this study, ADL (with severe dysfunction or worse) and IADL (serious dependence) were significant independent risk factors for postoperative complications. Poor functional status has also been reported to be associated with nursing home placement [46]. Since assessment of functional status can be performed even in a busy preoperative setting to obtain valuable information, it should be routinely included in the preoperative assessment. Nutritional status is extremely important for older patients undergoing surgery. Multiple scoring systems have been developed to assess nutritional status, which include subjective global assessment, the Nutritional Risk Index, the MNA-short form, and the Maastricht Index. The European Society for Clinical Nutrition and Metabolism advocates the use of the MNA as a screening tool, while members of the American Society for Parenteral and Enteral Nutrition do not recommend any one screening tool over another [47]. No matter which tool is chosen, malnutrition is believed to be associated with postoperative complications, especially severe infection [48]. As reported in previous studies, the severity of renal disease is associated with higher rates of postoperative complications, especially those patients with end-stage renal disease requiring hemodialysis [49, 50]. The use of a large nationwide database to compare outcomes between renal transplant and dialysis patients with diabetes after total hip arthroplasty found that renal dialysis was associated with increased risk of complication within 30 days after surgery [51]. In this study, renal function with CKD ≥ stage 3a was found to be independently associated with increased risk of postoperative complications.

There were several limitations to this study. First, the number of patients was relatively small, which may have caused statistical bias. Second, emergency surgery was excluded because of the lack of time to perform CGA in clinical practice. However, almost all spinal surgeries and total knee replacements were elective procedures. Third, because of the small number of patients, we were unable to establish a connection between specific postoperative complications and the preoperative CGA results. Fourth, postoperative rehabilitation exercise is an important part of CGA, postoperative rehabilitation exercises are guided by spine surgeons in our CGA team. Compared with physiotherapists, spine surgeons lack more professional postoperative rehabilitation guidance and treatment. Our CGA team is ready to add physiotherapists in future work.

## Conclusion

The CGA process reduces patient mortality and increases safety in older orthopedic surgery patients. The results of this study will allow orthopedic surgeons to better counsel their patients on the risks of postoperative complication. Spinal fusion, blood loss, ADL (severe dysfunction or worse), IADL (serious dependence), renal function (CKD ≥ stage 3a), and nutritional status (MNA, malnourished) were independent risk factors for postoperative complications in older patients following orthopedic surgery.

## Human and animal rights

This study has been performed in accordance with the ethical standards as laid down in the 1964.

Declaration of Helsinki and its later amendments or comparable ethical standards. No animals have.

been involved.

## Supplementary Information

**Additional file 1.**

## Data Availability

All data generated or analysed during this study are included in this published article [and its supplementary information files].
