# Supplementary material for: Comprehensive geriatric assessment for older orthopedic patients and analysis of risk factors for postoperative complications
Source: BMC Geriatr. 2022 Aug 4;22:644. doi: 10.1186/s12877-022-03328-5 (PMC9354431; doi:10.1186/s12877-022-03328-5)
Supplement: Supplementary file 1 — Additional file 1. [file 12877_2022_3328_MOESM1_ESM.docx]

**Table 5. Univariate Analysis of Preoperative CGA Related to Postoperative Complications**

|  | With any complication | p-value^a^ |  | With any complication | p-value^a^ |
| --- | --- | --- | --- | --- | --- |
| **ADL** |  | **0.0296 ^b^** | **Renal function** |  | **0.0134 ^b^** |
| independent | 21(19.0%) |  | normal | 7(0%) |  |
| mild dysfunction | 58(23.7%) |  | stage 1, | 22(22.7%) |  |
| moderate dysfunction | 75(28.4%) |  | stage 2 | 84(23.8%) |  |
| severe dysfunction | 45(39.5%) |  | stage 3a | 69(44.9%) |  |
| extremely severe dysfunction | 15(58.8%) |  | stage 3b | 26(38.5%) |  |
| **VAS** |  | 0.3976 | stage 4 | 4(0%) |  |
| no pain | 41(31.7%) |  | stage 5 | 2(0%) |  |
| mild pain | 75(26.7%) |  | **Child-Pugh** |  | 0.6248 |
| moderate pain | 33(24.2%) |  | grade A | 201(31.3%) |  |
| severe pain | 46(34.8%) |  | grade B | 7(14.3%) |  |
| very severe pain | 19(47.4%) |  | grade C | 6(33.3%) |  |
| **IADL** |  | **0.0354 ^b^** | **Cardiac risk** |  | 0.3314 |
| normal | 34(17.6%) |  | low risk | 139(33.1%) |  |
| mild dependence | 39(25.6%) |  | high risk | 75(26.7%) |  |
| moderate dependence | 80(28.8%) |  | **Respiration risk** |  | 0.1000 |
| serious dependence | 61(44.3%) |  | 0.5% | 49(32.7%) |  |
| **Frailty** |  | 0.4054 | 1.8% | 88(25.0%) |  |
| normal | 17(17.6%) |  | 4.2% | 68(41.2%) |  |
| prefrail | 55(29.1%) |  | 10.1% | 9(0%) |  |
| frail | 142(33.1%) |  | **Caprini thrombus risk** |  | 0.4681 |
| **Nutrition MNA** |  | **0.0442 ^b^** | moderate | 3(0%) |  |
| normal | 114(23.7%) |  | high | 15(26.7%) |  |
| at risk of malnutrition | 70(37.1%) |  | extremely high | 196(31.6%) |  |
| malnourished | 30(43.3%) |  | **Massive hemorrhage risk** |  | 0.1497 |
| **Perioperative blood glucose** |  | 0.7418 | low risk | 38(21.1%) |  |
| met the requirement | 191(31.4%) |  | high risk | 176(33.0%) |  |
| failed the requirement | 17(35.3%) |  | **Cognitive impairment** |  | **0.0052 ^b^** |
| diabetic ketosis | 3(0%) |  | no impairment | 101(19.8%) |  |
| hypoglycemia | 3(0%) |  | mild impairment | 113(40.7%) |  |
| **Thyroid disease and function** |  | 0.6740 | **Anxiety** |  | 0.6536 |
| Normal | 186(30.6%) |  | no anxiety | 209(30.6%) |  |
| hypothyroidism | 11(45.5%) |  | has anxiety | 5(40.0%) |  |
| Subclinical hypothyroidism | 13(30%) |  | **Depression** |  | 0.6536 |
| subclinical hyperthyroidism | 4(25%) |  | no depression | 209(30.6%) |  |
| **Stroke risk** |  | 0.8628 | had depression | 5(40.0%) |  |
| low | 98(32.7%) |  | **ASA score** |  | 0.0597 |
| moderate | 74(29.7%) |  | grade II | 39(16.7%) |  |
| high | 42(28.6%) |  | grade III | 163(35.3%) |  |
| **Fall risk** |  | 0.8678 | grade IV | 12(25.0%) |  |
| slight | 11(27.3%) |  | **Delirium risk** |  | 0.3127 |
| mild | 71(33.8%) |  | mild | 15(13.3%) |  |
| Moderate | 121(28.9%) |  | moderate | 183(32.2%) |  |
| high | 11(36.4%) |  | high | 16(31.3%) |  |
| **Polypharmacy risk** |  | 0.6465 |  |  |  |
| without risk | 54(33.3%) |  |  |  |  |
| with risk | 160(30.0%) |  |  |  |  |

Abbreviations: ADL, Activities of Daily Living; IADL, Instrumental Activities of Daily Living; MNA, Mini nutritional assessment; VAS, visual analogue scale; ASA, American Standards Association scale.

^a^, Kruskale-Wallis test.

^b^, Significant differences were found among groups.
